# Septic Shock in Hematological Malignancies: Role of Artificial Intelligence in Predicting Outcomes

**DOI:** 10.3390/curroncol32080450

**Published:** 2025-08-10

**Authors:** Maria Eugenia Alvaro, Santino Caserta, Fabio Stagno, Manlio Fazio, Sebastiano Gangemi, Sara Genovese, Alessandro Allegra

**Affiliations:** 1 Division of Hematology, Department of Human Pathology in Adulthood and Childhood “Gaetano Barresi”, University of Messina, Via Consolare Valeria, 98125 Messina, Italy; m.eugenia983@gmail.com (M.E.A.); santino.caserta@polime.it (S.C.); manliofazio@hotmail.it (M.F.); aallegra@unime.it (A.A.); 2 Allergy and Clinical Immunology Unit, Department of Clinical and Experimental Medicine, University of Messina, Via Consolare Valeria, 98125 Messina, Italy; gangemis@unime.it; 3 Institute for Biomedical Research and Innovation (IRIB), National Research Council of Italy (CNR), 98164 Messina, Italy; sara.genovese@irib.cnr.it

**Keywords:** septic shock, septic shock outcomes, infections, antibiotics, artificial intelligence, bundles, hematological malignancies

## Abstract

Septic shock is a life-threatening complication of sepsis, particularly in patients with hematologic diseases who are highly susceptible to it due to profound immune dysregulation. Recent advances in artificial intelligence offer promising tools for improving septic shock diagnosis, prognosis, and treatment in this vulnerable population. In detail, these innovative models analyzing electronic health records, immune function, and real-time physiological data have demonstrated superior performance compared to traditional scoring systems such as Sequential Organ Failure Assessment. In patients with hematologic malignancies, machine learning approaches have shown strong accuracy in predicting the sepsis risk using biomarkers like lactate and red cell distribution width, the latter emerging as a powerful, cost-effective predictor of mortality. Deep reinforcement learning has enabled the dynamic modelling of immune responses, facilitating the design of personalized treatment regimens helpful in reducing simulated mortality. Additionally, algorithms driven by artificial intelligence can optimize fluid and vasopressor management, corticosteroid use, and infection risk. However, challenges related to data quality, transparency, and ethical concerns must be addressed to ensure their safe integration into clinical practice. Clinically, AI could enable earlier detection of septic shock, better patient triage, and tailored therapies, potentially lowering mortality and the number of ICU admissions. However, risks like misclassification and bias demand rigorous validation and oversight. A multidisciplinary approach is crucial to ensure that AI tools are implemented responsibly, with patient-centered outcomes and safety as primary goals. Overall, artificial intelligence holds transformative potential in managing septic shock among hematologic patients by enabling timely, individualized interventions, reducing overtreatment, and improving survival in this high-risk group of patients.

## Simple Summary

Septic shock is a dangerous condition due to a severe reaction to infections, especially in people with blood cancers. These patients are more at risk because their immune systems are often very weak. New tools based on artificial intelligence can analyze medical records, body signals, and immune system activity, predicting who is likely to get worse and what treatments might work best. Certain blood tests, like those determining the amount of lactate or changes in red blood cells, can now be used to predict who is most at risk of dying. Some systems can even suggest the best choices of treatment based on how the patient’s body is reacting. Although there are still concerns about how these systems use data and make decisions, this technology could help physicians act faster and more accurately, leading to better chances of survival for very sick patients.

## 1. Introduction

### 1.1. General Considerations on Septic Shock

Septic shock is a critical, life-threatening condition that constitutes the most severe end of the sepsis spectrum. It is defined as a subset of sepsis and is characterized by circulatory, cellular, and metabolic instability linked to a higher risk of death than sepsis itself. According to the Third International Consensus Definitions for Sepsis and Septic Shock (2016), septic shock is characterized by persisting hypotension that requires vasopressors to maintain a mean arterial pressure (MAP) ≥65 mmHg and a serum lactate level >2 mmol/L (18 mg/dL), despite adequate volume resuscitation and a suspected or confirmed infection [1,2,3,4].

Sepsis affects around 50 million people globally every year, with a mortality that remains high (25–50%), even in high-income countries. In intensive care units (ICUs), sepsis occurs in up to 54.7% of the patients, with septic shock affecting approximately 15.8% of them. Septic shock is not just an advanced stage of sepsis but is a complex, dysregulated immune response to infection that causes acute circulatory failure and tissue hypoperfusion [5,6].

**Figure 1:**
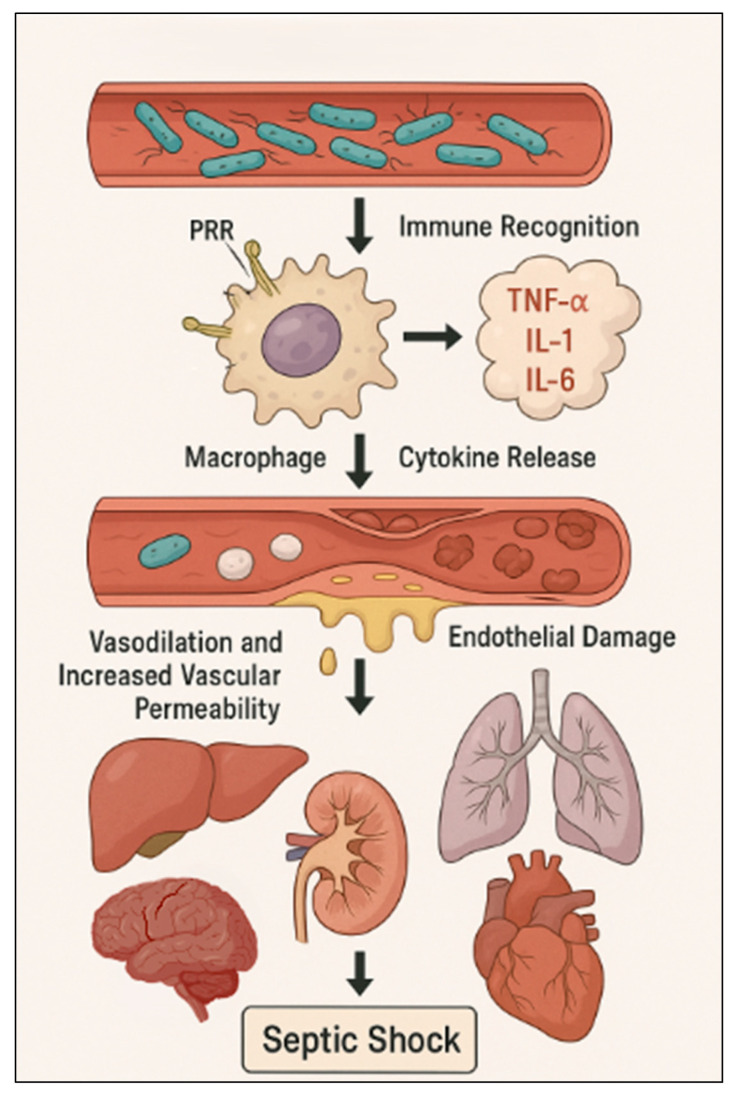
Pathogenesis of septic shock. PRR: pattern recognition receptor.

The condition starts with microbial invasion, triggering an excessive immune reaction through recognition of pathogen- and damage-associated molecular patterns (PAMPs and DAMPs) via pattern recognition receptors like Toll-like receptors (TLRs). This leads to a massive release of cytokines such as TNF-α, IL-1β, IL-6, and interferons, driving systemic inflammation, endothelial damage, and microcirculatory dysfunction. Increased vascular permeability and vasodilation result in hypotension and reduced tissue perfusion (Figure 1) [7,8,9,10].

Septic shock also causes a hypercoagulable state, which may progress to disseminated intravascular coagulation (DIC), leading to microvascular thrombosis and bleeding. Additionally, mitochondrial dysfunction and oxidative stress impair ATP production, causing cytopathic hypoxia. Elevated lactate levels signal tissue hypoperfusion. These interconnected mechanisms culminate in multiple organ dysfunction syndrome (MODS), affecting organs like the kidneys, liver, lungs, and heart [11,12,13,14].

### 1.2. Diagnosis and Risk Stratification of Septic Shock

A timely and accurate diagnosis of septic shock is critical, as delayed treatment significantly increases mortality. A comprehensive approach combining clinical assessment, biomarkers, hemodynamic monitoring, and advanced diagnostics is essential [15].

The initial suspicion of sepsis is based on clinical signs like fever, leukocytosis, or leukopenia, with a potential infection source. Hemodynamic compromise includes hypotension, tachycardia, altered mental status, and low urine output. Serum lactate >2 mmol/L indicates poor prognosis, with serial measurements tracking resuscitation. Inflammatory markers like C-reactive protein (CRP) and procalcitonin (PCT) support diagnosis establishment and guide antibiotic choice. Organ dysfunction markers confirm the diagnosis, while hemodynamic monitoring and tools like stroke volume variation guide therapy [16].

Presepsin, a novel biomarker, reflects early immune activation in bacterial infections. Presepsin levels rise early in sepsis, correlate with disease severity and prognosis, and may outperform CRP and PCT. Serial measurements may track treatment response, though their clinical utility requires further validation [17].

To identify the source of infection is of crucial importance. Imaging (X-ray, computed tomography, ultrasound, echocardiography) and microbiological tests (blood and site-specific cultures) enhance diagnostic precision. Modern diagnostics, including polymerase chain reaction (PCR), next-generation sequencing, and point-of-care testing (e.g., lactate, PCT), accelerate pathogen identification and support rapid clinical decision-making [18,19,20,21].

The early identification of sepsis in non-ICU settings can be aided by the quick Sequential Organ Failure Assessment (qSOFA) score, which is based on altered mentation, systolic BP ≤100 mmHg, and respiratory rate ≥22/min. A score ≥2 suggests higher risk. However, the full Sequential Organ Failure Assessment (SOFA) score, assessing dysfunction across six systems, is superior for critically ill patients. Septic shock is confirmed by persistent hypotension requiring vasopressors and lactate >2 mmol/L despite fluid resuscitation. Higher SOFA scores correlate with increased mortality [22,23,24].

In ICU settings, SOFA is crucial for diagnosing and monitoring septic shock, while qSOFA is used for screening in general wards. Other scores like Acute Physiologic Assessment and Chronic Health Evaluation II (APACHE II) and Simplified Acute Physiology II (SAPS II) estimate mortality but are less specific for sepsis. Severe organ failures and comorbidities worsen the prognosis, while artificial intelligence-driven prediction models show promise for personalized critical care [25,26,27,28].

### 1.3. How to Manage Septic Shock

Septic shock management focuses on rapidly restoring tissue perfusion with fluid resuscitation and vasopressor support. Current guidelines recommend administering an initial 30 mL/kg bolus of intravenous crystalloids, with balanced crystalloids like lactated Ringer’s preferred over normal saline. Fluid therapy should be guided by dynamic markers of responsiveness, such as passive leg raise testing, rather than by static measures like central venous pressure. If hypotension persists despite adequate fluid loading, norepinephrine is the first-line vasopressor, with vasopressin or epinephrine as adjuncts when necessary [29,30].

Timely antimicrobial therapy is critical in the management of septic shock, particularly in immunocompromised patients, such as those with hematologic malignancies. Broad-spectrum antibiotics should be administered ideally within one hour of clinical diagnosis to reduce mortality. Blood and other relevant cultures must be obtained prior to initiating antibiotics to allow for pathogen identification and targeted therapy. Subsequent de-escalation of antibiotics based on culture results and clinical response is essential to minimize resistance and toxicity. In parallel, source control measures—such as abscess drainage, removal of infected devices, or surgical intervention—should be initiated within 6 to 12 h, as delays are associated with worse clinical outcomes [30,31].

Low-dose hydrocortisone may help in refractory septic shock, though its routine use is not recommended. Glycemic control should maintain blood glucose below 180 mg/dL. For patients with acute respiratory distress syndrome (ARDS), lung-protective ventilation is essential. Continuous monitoring, including serial lactate measurements and advanced hemodynamic assessments, is crucial to guide therapy. Renal replacement therapy may be needed for sepsis-induced acute kidney injury. Future strategies may involve precision medicine, immunotherapy, and experimental therapies like cytokine removal [29,31].

### 1.4. Septic Shock in Haematological Disorders

Patients with hematological malignancies represent a highly vulnerable population in the context of sepsis and septic shock, due to disease-related immune dysfunction and the immunosuppressive effects of chemotherapy, corticosteroids, and hematopoietic stem cell transplantation (HSCT). Epidemiological studies indicate that up to 25–30% of these patients may develop septic shock during hospitalization, particularly those undergoing intensive chemotherapy or hematopoietic stem cell transplantation. Gram-negative bacteria, especially *Pseudomonas aeruginosa*, and multidrug-resistant organisms are common pathogens. The risk is heightened in neutropenic individuals, with mortality rates exceeding 50% in some cohorts [32].

Clinical recognition of sepsis and septic shock in these patients is often delayed. Fever may be absent in neutropenic patients, while inflammatory markers are unreliable due to underlying cytopenias. As a result, septic shock frequently manifests abruptly, often within the first two weeks following chemotherapy, during profound neutropenia and mucosal barrier injury. Infections in hematological patients are often caused by Gram-negative bacilli, especially multidrug-resistant (MDR) pathogens. Silva and colleagues found that *Klebsiella pneumoniae* and *Pseudomonas aeruginosa* were among the most common causative agents of septic shock in neutropenic patients. Invasive fungal infections, particularly due to *Candida* spp. and *Aspergillus*, further complicate sepsis management and are strong predictors of poor prognosis. The presence of MDR organisms, delays in initiating appropriate antimicrobial therapy, and persistent neutropenia were independently associated with increased 30-day mortality. These pathophysiologic changes facilitate microbial translocation and bloodstream infection, increasing the risk of severe sepsis and shock [33]. Of particular concern are patients undergoing allogeneic HSCT, who face exacerbated risks due to graft-versus-host disease (GVHD) and prolonged immunosuppression [34].

Hemodynamic resuscitation strategies in these patients also require careful individualization. Bozkurt and colleagues observed that fluid overload—often due to excessive resuscitation—and delayed vasopressor initiation contributed to increased mortality. Early goal-directed therapy may be non-optimal in hematological patients; rather, early vasopressor support with judicious fluid administration appears to improve outcomes [35].

Since Islaz-Munoz confirmed that the ICU mortality of septic hematological patients significantly exceeds that of those with solid tumors, the need for proactive ICU referral, early source control, and rapid antimicrobial therapy appears crucial [36]. Moreover, recent epidemiological data suggest that many prognostic factors remain poorly defined in this population and that prospective studies are urgently needed to refine diagnostic and therapeutic strategies [32,37].

In the context of hematological malignancies, acute myeloid leukemia (AML), characterized by bone marrow failure and resulting neutropenia, makes patients highly vulnerable to infections, which are further exacerbated by myelosuppressive treatment aimed at achieving remission. The progression from febrile neutropenia (FN) to septic shock is often rapid, with a median interval of just 1.6 months from AML diagnosis to the onset of shock. In fact, septic shock complicates the hospital admission for AML in a third of patients. The prompt development of septic shock, typically within a day of FN diagnosis, highlights the urgency of recognizing this complication early and initiating appropriate interventions without delay. A study performed in a single center over three years examined the outcomes of AML patients who developed septic shock and were treated according to Surviving Sepsis Campaign (SSC) guidelines. Of the 37 patients included in the cohort, most presented with FN, and the majority had multi-organ dysfunction, with the cardiovascular, respiratory, and renal systems being most affected. The etiological agents responsible for septic shock were primarily bacterial, with *Klebsiella* spp., *E. coli*, and *Pseudomonas aeruginosa* being the most frequent pathogens isolated in cultures. Despite the early initiation of empiric antibiotics, which included agents such as meropenem and cefepime, the mortality rate associated with septic shock in this cohort was high. The median overall survival after the diagnosis of septic shock was only nine days, with nearly 70% of the patients dying because of septic shock. Notably, some patients who survived beyond the resolution of cardiovascular dysfunction still faced poor outcomes due to the progression of their underlying disease or subsequent infectious complications. The study underscores the importance of aggressive treatment, including the use of vasopressor support with noradrenaline, but also highlights the poor prognosis for this patient population. In conclusion, septic shock in AML patients represents a rapid progressive and highly fatal complication, with a median survival of just over a week after the onset of shock. While adherence to the SSC guidelines is critical in managing these patients, the study indicates that the prognosis remains the same, despite early recognition and intervention. The findings underscore the need for continued efforts to improve sepsis management and survival outcomes in this particularly vulnerable patient group [38].

In conclusion, septic shock in patients with hematological malignancies presents distinct pathophysiological features, microbiological profiles, and therapeutic challenges. Prompt recognition, early ICU-level care, and individualized antimicrobial and hemodynamic strategies are essential to improve survival in this high-risk group.

## 2. Artificial Intelligence

### 2.1. General Background

Artificial intelligence (AI) refers to the capability of machines and computer systems to mimic human intelligence and cognitive functions. These systems are programmed to perform tasks that typically require human reasoning, such as analyzing data, solving problems, learning from experience, understanding natural language, recognizing patterns, and making complex decisions. Unlike traditional software, which follows a fixed set of instructions, AI systems are designed to adapt and evolve based on new data and interactions with their environment [39].

AI can be categorized into three main types: narrow AI, general AI, and artificial superintelligence (ASI). Narrow AI, or weak AI, is designed to perform specific tasks and is the most used form today. Examples include voice assistants like Siri and Alexa, image recognition software, and recommendation systems used by platforms like Netflix and YouTube. These systems operate efficiently within defined boundaries but lack broader reasoning ability or awareness.

General AI, or strong AI, is a theoretical concept implying machines capable of understanding, learning, and applying knowledge across various tasks—like human intelligence. It would be capable of reasoning, problem-solving, and emotional understanding. Though it does not yet exist, it remains a key research goal.

Artificial superintelligence goes beyond human intelligence, excelling in all areas including creativity and social reasoning. Though speculative, ASI raises ethical and existential concerns, emphasizing the need for responsible AI governance [40,41].

Key technologies behind AI include machine learning (ML) and deep learning (DL). ML allows systems to learn from data and improve over time, while DL, using neural networks, models complex patterns and powers advancements in areas like speech recognition and autonomous vehicles. Other vital components include natural language processing (NLP) and computer vision, enabling AI to understand language and analyze visual data, making it adaptable across various real-world applications (Table 1) [42,43,44,45,46,47,48,49].

### 2.2. Focus on AI in Healthcare and Medicine

AI is transforming healthcare, particularly in diagnostics and medical imaging. AI algorithms, especially those based on deep learning, can analyze X-ray images, magnetic resonance imaging (MRI) findings, and computer tomography (CT) scans with accuracy that rivals or surpasses that of experienced radiologists [50]. These systems identify conditions like cancers, fractures, pulmonary embolisms, and retinal diseases, reducing human error and enhancing the diagnostic precision. AI models, such as those developed by DeepMind and companies like Aidoc, assist in real-time clinical workflows, improving efficiency [51,52].

AI also plays a significant role in predictive analytics by examining patient data to forecast disease progression and clinical outcomes. By identifying subtle patterns, AI helps healthcare providers detect high-risk patients earlier, triggering early warning systems in ICUs to anticipate patient deterioration before traditional signs appear. Additionally, AI models predict hospital readmissions, optimizing resource allocation [53,54].

In drug discovery, AI accelerates the traditionally long and costly process. Machine learning identifies promising drug candidates by analyzing molecular structures and biological data, reducing the need for lab testing. AI also aids in repurposing existing drugs for new uses, making the process faster and less risky. Companies like Atomwise and BenevolentAI are leading this shift.

AI supports personalized medicine by analyzing genetic and lifestyle data to tailor treatments to individual patients. In oncology, AI recommends the most effective therapies based on tumor genetics, improving outcomes and reducing side effects. AI-powered virtual healthcare tools, such as medical chatbots, enhance accessibility and reduce strain on healthcare systems. In robotic surgery, AI provides surgeons with precision and real-time data, leading to less invasive procedures and quicker recovery times [55,56].

AI transparency and interpretability are critical in clinical settings, especially when applied to high-risk populations such as patients with hematologic malignancies. Clinicians must understand how AI models generate outputs to ensure informed decision-making, trust, and accountability. Black-box algorithms—common in deep learning—pose challenges by obscuring the reasoning processes, which potentially limits their clinical adoption. Transparent, interpretable AI systems that provide rationale for predictions, such as risk scores or suggested interventions, are essential for integration into sepsis management. Enhancing model explainability not only supports regulatory compliance and ethical standards but also facilitates multidisciplinary collaboration and safe, personalized care in complex scenarios like septic shock.

In Table 2, most of the applications of AI in medicine are presented.

## 3. The Use of AI in Septic Shock

Septic shock is associated with high mortality and demands prompt intervention to improve patient outcomes. In recent years, artificial intelligence, particularly through machine learning and deep learning algorithms, has demonstrated growing potential in transforming septic shock management. AI can analyze large volumes of complex clinical data in real time, enabling an earlier and more accurate detection of sepsis onset before overt clinical deterioration occurs. Additionally, AI-driven models can support refined risk stratification by identifying subtle patterns and predictive biomarkers often missed by conventional methods. These tools also facilitate the design of individualized treatment strategies, including dynamic fluid management, antimicrobial optimization, and vasopressor titration, ultimately enhancing patient care [64].

Traditional scoring systems such as qSOFA and SOFA rely on clinical judgment and often lack sufficient sensitivity for timely detection. AI models trained on large-scale electronic health record (EHR) data address these limitations. In a study by Liu and colleagues, machine learning algorithms were used to analyze large datasets from EHRs, including vital signs, lab results, and patient demographics. By applying ML techniques such as random forests and deep neural networks, the model was able to predict the onset of sepsis earlier than conventional methods. The AI algorithm demonstrated a high performance, achieving an area under the receiver operating characteristic curve (AUROC) of 0.91, highlighting its potential for early detection. Prompt sepsis identification allows for earlier therapeutic actions, which enhance the survival rates and decrease the risk of adverse outcomes [65,66].

Similarly, Deng and colleagues created a predictive model for ICU patients that achieved an area under the curve (AUC) of 0.82 for sepsis and 0.84 for septic shock, with 79.1% sensitivity and 73.3% specificity. Their work emphasized the importance of using diverse datasets and real-time EHR processing to build robust, generalizable prediction tools across different ICUs [67,68].

Zhang and his group utilized time-series data from ICU monitors alongside static clinical variables to predict sepsis progression. Their AI model reached an AUROC of 0.88, exceeding the predictive accuracy of qSOFA and SOFA and enabling the early identification of patients at risk of developing septic shock [69].

Risk stratification is equally crucial for guiding clinical decisions. Zhang and colleagues applied ML to estimate the mortality risk among septic shock patients using indicators such as organ dysfunction scores, biomarkers, and hemodynamic parameters. Their model effectively distinguished high- and low-risk patients, supporting more targeted therapeutic approaches [70]. Yang demonstrated that ML algorithms outperformed SOFA in predicting both septic shock and mortality by incorporating structured and unstructured EHR data [71].

Regarding treatment, AI has been instrumental in optimizing fluid resuscitation and vasopressor therapy. Wang et al. developed a model that personalized fluid administration using dynamic physiological data, reducing both under- and over-resuscitation and improving patient outcomes [72]. Gupta and colleagues designed an AI tool, based on continuous data monitoring, to guide the initiation and titration of vasopressors like norepinephrine, improving hemodynamic control and reducing adverse effects [73,74].

Among the most promising AI approaches nearing clinical implementation in septic shock management for hematologic patients are ML models using structured EHR data for early sepsis prediction and risk stratification. Gradient boosting algorithms and random forest classifiers have demonstrated robust performance in identifying high-risk patients using vital signs, laboratory markers, and treatment history. These models are increasingly integrated into clinical decision support systems. In contrast, experimental techniques such as deep reinforcement learning and immune system digital twins remain in preclinical phases, offering potential for personalized therapy modeling but requiring further validation before routine clinical application.

Therefore, AI systems integrated with bedside monitors and wearable sensors, as shown by Liu, enable continuous surveillance by tracking variables such as lactate levels, oxygen saturation, and heart rate variability. These systems provide real-time alerts, allowing for timely interventions and improving patient safety. However, their successful clinical integration requires effective communication between AI tools and clinicians to ensure trust and usability [75,76].

### 3.1. AI in Patients with Septic Shock and Hematological Diseases

Septic shock in patients with hematologic disorders is a particularly challenging clinical scenario, characterized by profound immune dysregulation, an increased susceptibility to infections, and a high mortality rate. Recent advancements in AI have introduced promising technologies that are poised to improve diagnostic accuracy, enhance therapeutic decision-making, and optimize outcomes for these patients [77]. A study by Tahir explored the application of ML algorithms to classify the lactate levels and assess the sepsis risk based on neutrophil phagocytic activity. A clustergram analysis revealed key differences in phagocytic activity between control and high-risk patient groups, with darker shades in the heatmaps indicating higher bead engulfment and suggesting increased phagocytic activity in high-risk patients. Machine learning models were applied to both the original and a pruned dataset (with up to two outliers removed) to improve classification accuracy and reduce overfitting. Due to the small sample size, simpler ensemble-based models—bagged decision trees (DTs), k-nearest neighbors (k-NNs), and naïve Bayes (NB)—were selected, and each was trained 100 times with data shuffling.

Among these, the ensemble bagged decision tree performed best on the pruned data, achieving an average accuracy of 78.3% and an AUC of 0.782. In contrast, its performance on the original data was lower. While k-NN and NB showed slightly lower accuracy, they achieved higher AUC values due to class imbalance favoring high-risk cases.

Although the false positive and false negative rates remained high due to the limited data, the overall model performance improved with data pruning. The study demonstrates that machine learning can help distinguish sepsis risk levels, but large, comprehensive datasets—integrating clinical information—are essential for developing reliable diagnostic tools for clinical application [78].

Sepsis is a major cause of thrombocytopenia in the ICU, which is associated with higher mortality and prolonged organ support. Red cell distribution width (RDW), an indicator of red blood cell variability, was found to be strongly associated with mortality in septic shock patients with thrombocytopenia; in fact, an elevated RDW likely reflects intense inflammation and oxidative stress, which can increase the release of immature red blood cells into the circulation. A retrospective cohort study by Ling and colleagues examined the association between RDW and clinical outcomes in septic shock patients with thrombocytopenia. The study found that mortality was significantly higher in patients with thrombocytopenia upon admission. Logistic regression analysis identified age, RDW, lactate, and SOFA score as independent predictors of death. The Shapley additive explanations-based (SHAP-based) interpretation of an extreme gradient boosting (XGBoost) model revealed that RDW was the second most important predictor of 28-day mortality, following the SOFA score. The RDW maintained its predictive value even after adjustment for covariates using propensity score matching (PSM), with receiver-operating characteristic curve analysis demonstrating its superior predictive ability compared to other factors [79]. This study highlights RDW as a simple, reliable, and inexpensive predictor of mortality in this patient group, though the findings need further validation through multi-center prospective studies.

In patients with septic shock and underlying hematologic disease, prolonged cytopenias and recurrent infections are common, particularly during neutropenia. Standard management often includes hospitalization, antifungal prophylaxis, and administration of granulocyte colony-stimulating factor (G-CSF) to mitigate the infection risk. However, predicting which patients will develop prolonged cytopenias or severe infections remains challenging. AI tools analyzing longitudinal blood counts, microbiologic data, and treatment history could enhance the early identification of high-risk profiles, enabling tailored prophylactic strategies and optimized resource allocation. Incorporating AI in this context may improve patient outcomes by anticipating complications and guiding personalized supportive care during immunosuppressive phases.

Current sepsis management guidelines, including those for septic shock, are largely based on studies in the general population and lack specificity for patients with hematologic malignancies. These individuals present distinct challenges due to profound immunosuppression, atypical clinical presentations, and higher rates of multidrug-resistant infections. Standard protocols may not account for delayed inflammatory responses, cytopenias, or the influence of recent chemotherapy. As a result, risk stratification, antimicrobial selection, and supportive interventions are often suboptimal. These limitations highlight the need for AI-driven approaches that integrate high-dimensional clinical, laboratory, and treatment data to provide real-time, personalized decision support tailored to hematologic patients’ unique profiles.

Moreover, a different study demonstrated the potential of deep reinforcement learning (DRL) in simulating immune responses and adapting treatment protocols for septic shock. To improve sepsis diagnosis, researchers applied machine learning to gene expression data from peripheral blood. They previously identified six downregulated genes (6-HubGss) in septic shock and sepsis patients. These genes distinguished patients from healthy individuals but could not differentiate sepsis from septic shock. To address this issue, they developed SepxFindeR, an ensemble machine learning model using LASSO regression on combined data from three datasets (GSE95233, GSE57065, and GSE154918). SepxFindeR outperformed previous models (LDA6-HubGss and RSA6-HubGss), showing high accuracy in identifying septic shock and sepsis and significantly improvement in distinguishing between the two conditions.

SepxFindeR was then tested on an independent RT-qPCR dataset of 46 participants (15 sepsis, 13 septic shock, and 18 healthy individuals). Although 6-HubGss expression was lower in both patient groups compared to the controls, there was no expression difference between sepsis and septic shock patients. LDA and RSA models again failed to distinguish between them. In contrast, SepxFindeR correctly identified all sepsis and septic shock cases and differentiated them with 92.9% accuracy. These results suggest that SepxFindeR is a robust, high-performing tool for detecting and classifying sepsis conditions, including rapid RT-qPCR-based diagnostics, with potential for clinical application [80].

The work by Sung and colleagues further supports the use of AI in sepsis management, specifically focusing on pediatric patients with septic shock and hematologic conditions. In their study, the researchers used machine learning-based models to predict the effects of corticosteroid treatments, showing improved clinical outcomes compared to traditional scoring systems like SAPS II. Pediatric hematological patients are particularly vulnerable to septic shock, and traditional sepsis scoring systems are not always optimized for this population. The application of personalized treatment algorithms using ML could provide clinicians with more tailored recommendations, reducing the risk of overtreatment or undertreatment and leading to better overall outcomes. The ability to predict how a patient might respond to corticosteroids based on their individual clinical and genetic profile marks a significant leap toward precision medicine in the management of sepsis [81].

Finally, a recent paper introduced a predictive immunological AI model to identify early signs of immunosuppression and infection risk in septic patients with hematological diseases. This study demonstrates that TNFα production after LPS challenge is significantly lower in patients with sepsis and bacteremia compared to healthy individuals and those undergoing cardiac surgery. This reduction reflects endotoxin tolerance (ET), indicating innate immune suppression, and shows promise for TNFα as an early diagnostic biomarker for sepsis and bacteremia. The inability to produce TNFα upon LPS stimulation correlates with disease severity, and its evaluation could enhance current triage methods in emergency departments (EDs).

Existing diagnostic methods, such as vital sign assessment and standard blood tests, lack specificity and are influenced by treatments. While models based on clinical and laboratory data (e.g., CRP levels or flow cytometry parameters) have shown potential, they often lack validation or are ICU-specific. This study highlights the potential of ET evaluation, particularly of TNFα production, as a rapid and specific point-of-care test to identify high-risk patients and distinguish sepsis from non-complicated infections.

Moreover, standard bundles, as defined by the Surviving Sepsis Campaign, emphasize early recognition, administration of broad-spectrum antimicrobials within one hour, and vasopressor initiation for refractory hypotension.

Early warning algorithms embedded within the electronic health record (EHR) can continuously analyze multi-parametric data—including vital signs, laboratory trends, and dynamic risk scores—to detect subtle physiological deterioration. When predefined thresholds are reached, the system can automatically activate bundle-based interventions, such as notifying the rapid response team, prompting empiric antimicrobial initiation, or recommending point-of-care ultrasonography to guide fluid resuscitation. Integration with computerized physician order entry (CPOE) facilitates immediate order set activation, while dashboards provide real-time feedback on bundle completion and compliance.

Furthermore, AI can tailor bundle execution to the hematological population by incorporating patient-specific variables such as recent chemotherapy, stem cell transplant status, or antifungal prophylaxis. Decision support can prioritize empiric coverage for multidrug-resistant organisms or opportunistic infections and suggest hemodynamic strategies that minimize fluid overload in patients with anthracycline-induced cardiomyopathy. Embedding these AI-enabled decision points into multidisciplinary care ensures that bundle adherence is both timely and individualized. This approach may reduce variability in sepsis management, shorten the time to goal-directed interventions, and improve the survival outcomes in hematologic patients experiencing septic shock.

Furthermore, the ET test may help identify immunosuppressed patients before overt organ dysfunction occurs. Though promising, further validation is needed. This assay could complement rapid molecular diagnostics and improve antimicrobial stewardship. Ultimately, ET testing may become part of precision medicine strategies, guiding early diagnosis and tailored treatment in sepsis care [82].

Despite advances in critical care, significant gaps remain in the management of septic shock in patients with hematological malignancies. These include delayed diagnosis due to atypical presentations, difficulty in early risk stratification, and challenges in tailoring antimicrobial therapy amidst rising multidrug resistance. In this context AI could offer promising solutions, such as real-time predictive models for early sepsis detection, personalized risk assessment, and optimization of antimicrobial stewardship; it can also integrate vast clinical and laboratory data to support dynamic decision-making, potentially improving outcomes and reducing mortality in this complex and high-risk patient group.

Current clinical implementation of AI in septic shock detection among hematologic patients faces several barriers specific to hematology settings. These include the need for specialized ICUs equipped to manage profound immunosuppression, complex transplant-related complications, and prolonged cytopenias. Data heterogeneity from fragmented care across oncology, hematology, and intensive care units complicates AI model training and integration. Moreover, atypical infection presentations and rapid clinical deterioration challenge the standard input variables used in general sepsis models. Limited external validation in hematologic cohorts and lack of real-time interoperability with hospital systems further delay AI adoption, underscoring the need for tailored, context-aware AI solutions in this high-risk population.

Collectively, these studies illustrate the transformative potential of AI in managing septic shock in patients with hematologic diseases (Table 3). The use of AI in early diagnosis, immune response monitoring, personalized treatment planning, and infection risk prediction represents a paradigm shift in how sepsis is approached in this high-risk group. By integrating machine learning, deep learning, and reinforcement learning, healthcare providers can make more informed and timely decisions, potentially reducing the mortality associated with sepsis in these vulnerable patients. As the field continues to evolve, it is expected that AI will become an integral part of clinical practice, driving better outcomes through personalized, data-driven interventions and offering hope for improving the prognosis of patients with hematological diseases who develop septic shock.

### 3.2. Practical AI-Based Management of Septic Shock in Hematological Patients

To operationalize the integration of AI tools into the management of septic shock in patients with hematological malignancies, a structured clinical pathway can be envisioned. Upon hospital admission, early warning scores such as NEWS2 or qSOFA can be supplemented by AI-based risk stratification models using patient-specific variables (e.g., neutrophil count, prior chemotherapy, comorbidities) extracted from the electronic health record (EHR). Machine learning classifiers (e.g., XGBoost, random forest models) trained on multi-center sepsis datasets can provide the real-time prediction of sepsis onset or deterioration, prompting early antimicrobial administration and ICU transfer [66].

In the emergency department or ICU setting, predictive analytics platforms such as the epic sepsis model (ESM) or in-house AI dashboards can continuously monitor hemodynamic and laboratory parameters to detect subtle patterns of decompensation. Integration into clinical workflows is feasible through decision support alerts embedded in the EHR, which notify physicians when the model’s risk threshold is surpassed. For example, in neutropenic patients, real-time alerts linked to fever curves and biomarker kinetics (e.g., procalcitonin, CRP) may trigger pre-emptive broad-spectrum antibiotic coverage and additional surveillance [65].

Furthermore, federated learning approaches have shown promise in enabling AI models to learn from data across multiple institutions while preserving data privacy—crucial for rare oncologic cohorts. In practice, clinicians can participate in these networks by contributing anonymized data to improve model generalizability. Lastly, integration with hospital command centers and antimicrobial stewardship programs can optimize resource allocation and reduce inappropriate antibiotic use.

These use cases illustrate how the current AI technology is transitioning from research into applied clinical decision-making, with specific value in high-risk hematologic populations.

## 4. Future Perspectives

The future use of artificial intelligence in septic shock management holds great promise, particularly in enhancing early diagnosis, risk stratification, and personalized treatment. AI algorithms, especially machine learning and deep learning, can analyze vast datasets from EHRs, including vital signs, laboratory results, and genomic data to detect subtle patterns that may elude traditional clinical assessments. AI-driven predictive models can forecast septic shock hours before clinical signs appear, allowing for earlier intervention and improving outcomes. Real-time monitoring and decision support systems can alert clinicians to condition changes and suggest therapeutic adjustments [84,85].

The integration of AI into healthcare systems holds significant promise for improving the management of septic shock in patients with hematologic malignancies, a highly vulnerable and complex subgroup. AI-driven models can analyze vast amounts of clinical, laboratory, and genomic data to identify subtle patterns predictive of sepsis onset, support real-time risk stratification, guide antimicrobial selection, and optimize fluid and vasopressor management based on dynamic patient profiles. However, several challenges remain. Data quality and interoperability across institutions can limit model performance and generalizability. Incomplete or biased data can lead to inaccurate or unsafe predictions, particularly in underrepresented patient groups. Additionally, the “black box” nature of many AI algorithms raises concerns about interpretability, making it difficult for clinicians to trust or act on AI-generated recommendations. Ensuring transparency and compliance with data protection regulations such as the Health Insurance Portability and Accountability Act (HIPAA) and the General Data Protection Regulation (GDPR) is essential. Preventing algorithmic bias is equally critical to ensure equitable care for all patients. Moreover, successful AI integration requires clinician training, interdisciplinary collaboration, and robust regulatory frameworks to deploy AI tools safely and effectively in clinical practice [26,86].

Future strategies to overcome challenges in AI application for septic shock in hematologic patients must prioritize algorithmic fairness and transparency. Developing diverse, representative training datasets can reduce bias related to demographic and clinical heterogeneity in this population. Incorporating explainable AI techniques will enhance model interpretability, allowing clinicians to understand decision pathways and build trust. Continuous monitoring and validation in real-world clinical environments are essential to detect and correct emerging biases or errors. Multidisciplinary collaboration among hematologists, intensivists, data scientists, and ethicists will facilitate the creation of transparent, robust AI tools tailored to hematologic patients’ unique needs, ultimately improving their clinical outcomes.

Innovations in genomic and computational technologies are advancing risk stratification, with AI models outperforming traditional scoring systems like SOFA and APACHE II in predicting ICU mortality. However, achieving the clinical integration of AI tools requires overcoming hurdles in data quality, regulatory approval, and accountability. Regulatory bodies like the Food and Drug Administration (FDA) and the European Medicines Agency (EMA) must rigorously test AI systems before their deployment. As AI becomes more integral to healthcare, clear accountability frameworks are necessary to ensure patient safety and trust in these technologies [87,88].

## 5. Conclusions

The integration of artificial intelligence into the clinical management of septic shock represents a pivotal advancement, particularly for patients with hematological disorders who face disproportionately high risks of morbidity and mortality [1,78,89].

Patients with immune dysregulation often present atypically, complicating diagnosis and intervention. AI-driven models enable early detection, personalized treatment, and continuous monitoring using real-time data from electronic health records, lab results, and immunological parameters. Machine learning and deep reinforcement learning offer superior accuracy in predicting septic shock and guiding therapy compared to traditional scoring systems. Novel biomarkers, like RDW and neutrophil phagocytic activity, help predict outcomes. AI might also tailor treatment, improving fluid resuscitation and vasopressor and corticosteroid use, leading to better outcomes in pediatric hematology and optimized infection risk stratification in immunosuppressed patients [81,90].

Despite its benefits, challenges remain in fully implementing AI in clinical settings, such as data standardization, model interpretability, and algorithmic bias. Its real-time clinical use requires robust infrastructure, collaboration, and ongoing evaluation. Regulatory oversight and clinician training are vital for trust and optimal outcomes. AI shows transformative potential in critical care, particularly for vulnerable patients, improving decision-making and reducing the burden of sepsis.

Short-term recommendations for AI use in septic shock among hematologic patients focus on integrating validated predictive models into clinical workflows to enhance early diagnosis and risk stratification. Emphasis should be placed on improving data quality and ensuring model transparency to build clinician trust. Long-term efforts must prioritize developing adaptive, personalized AI systems that incorporate multi-omics and real-time monitoring, supported by robust multi-center validation and ethical frameworks, guiding precision medicine, and improving outcomes in this complex population.

Continued innovation and validation are essential for shaping the future of septic shock care, with AI supporting faster, safer, and more effective therapies [17,88].

## Figures and Tables

**Table 1:** Types of artificial intelligence and core AI components.

Category	Description	Examples	References
Weak AI	AI specialized in a single task; lacks general reasoning ability or awareness.	Siri, Alexa, Google Assistant, Netflix recommendations.	[40,41]
Strong AI	Hypothetical AI with human-like reasoning ability, learning, and emotional understanding across tasks.	Still theoretical; focus of academic research.	[40,41]
Artificial Superintelligence	AI surpassing human intelligence in all areas (science, creativity, etc.); raises ethical and existential concerns.	Speculative; discussed in AI safety and ethics literature.	[40,41]
Machine Learning (ML)	Algorithms that learn from data and improve over time without being explicitly reprogrammed.	Spam filters, fraud detection, predictive analytics.	[42,43]
Deep Learning (DL)	Subset of ML using artificial neural networks to model complex data; inspired by the human brain.	GPT models, AlphaGo, autonomous vehicles, diagnostic imaging.	[42,43]
Natural Language Processing (NLP)	Enables machines to understand and generate human language.	Language translation, chatbots, sentiment analysis.	[45]
Computer Vision	Allows machines to process and analyze visual information from the environment.	Facial recognition, object detection, medical image analysis.	[45]

**Table 2:** Main applications of AI in medicine.

Field	AI Application	Description	Reference
Radiology	Medical image analysis	AI used to interpret radiological images, enhance image quality, and assist in diagnosing various diseases.	[57]
Cardiology	Monitoring and diagnosis of cardiovascular diseases	AI models analyze ECGs, echocardiograms, and medical images to monitor and diagnose cardiovascular diseases.	[58]
Neurology	Diagnosis and prognosis of neurological diseases	AI to analyze brain images (such as MRI and CT scans) and develop predictive models for diseases like Alzheimer’s, Parkinson’s, and stroke.	[59]
Surgery	Planning and assistance in surgical procedures	AI to design personalized surgeries and assist in robot-assisted surgery during operations.	[60]
Precision Medicine	Personalized therapy and prediction of drug responses	AI to analyze genetic and clinical data for personalized treatment and prediction of drug responses.	[61]
Emergency Medicine	Risk prediction and automated triage	AI to analyze patient data and assist in triage processes and risk management in emergency situations.	[62]
Pharmacology	Drug development and monitoring	AI for accelerating drug discovery and analyzing pharmacological interactions and side effects.	[63]

**Table 3:** AI applications in septic shock in hematological conditions: objectives, study populations, and main findings.

Technology Used	Main Objective	Study Population	Key Findings	References
Machine Learning	Lactate risk classification based on neutrophil phagocytic activity	Patients with suspected sepsis	78% accuracy, AUC 0.78; potential use for early prediction	[78]
Deep Learning + Diagnostic AI	Evaluation of immune response efficacy in sepsis	Patients with hematologic disorders and fungal infections	Increased diagnostic accuracy for invasive infections through AI analysis	[79]
Deep Reinforcement Learning	Simulation of immune response and adaptive treatment	Simulated models	Reduced simulated mortality compared to standard therapy	[82,83]
Machine Learning	Personalized estimation of corticosteroid treatment effect	Pediatric patients with septic shock	Better clinical outcomes compared to standard models like SAPS II	[81]
Predictive Immunological AI	Early identification of immunosuppression and infection risk	Septic patients with hematologic diseases	Reliable prediction of positive cultures, useful for guiding therapy	[80]
